# Can a Mother's Parenting Style Predict Adolescent Oral Hygiene Behavior? A Self-Reported Cross-Sectional Study

**DOI:** 10.1055/s-0043-1768470

**Published:** 2023-06-19

**Authors:** Deva Annisa, Fitri Ariyanti Abidin, Arlette Suzy Setiawan

**Affiliations:** 1 Bachelor of Dentistry Study Program, Faculty of Dentistry, Universitas Padjadjaran, Bandung, Indonesia; 2 Department of General and Experimental Psychology, Faculty of Psychology, Universitas Padjadjaran, Bandung, Indonesia; 3 Department of Pediatric Dentistry, Faculty of Dentistry, Universitas Padjadjaran, Bandung, Indonesia

**Keywords:** supportive parenting style, nonsupportive parenting style, oral hygiene behavior, adolescents

## Abstract

**Objective** Adolescent oral hygiene behavior plays a crucial role into adulthood and still requires attention from parents, especially mothers. Parenting done by the mother will directly affect the child's life, including the child's oral hygiene behavior. The aim of this study was to determine the role of the mother's parenting style in predicting adolescent oral hygiene behavior.

**Materials and Methods** A quantitative study with a cross-sectional approach was used to examine the role of parenting style on their teenage child's oral hygiene behavior. The instruments used were (1) a parenting style measuring instrument to measure supportive and nonsupportive parenting styles of mothers (parent as social context questionnaire) and (2) a self-structured oral hygiene behavior measuring instrument based on the theory of planned behavior. The population of this study were students at SMP PGRI Depok and SMP Negeri 11 Depok, West Java, aged between 12 and 14 years. The sampling technique used total sampling on SMP PGRI students and multistage random sampling on students at SMP Negeri 11 Depok with a total sample of 230 students. Data analysis was done using multiple linear regression test.

**Results** Statistical regression tests showed that nonsupportive parenting style did not predict adolescent oral hygiene behavior (p = 0.567), while supportive parenting style did predict adolescent oral hygiene behavior ( *p* = 0.000). Supportive parenting style predicts 31.2% of adolescent oral hygiene behavior.

**Conclusion** Mother's supportive parenting style plays a role in predicting adolescent oral hygiene, and the mother's nonsupportive parenting style does not play a role in predicting adolescent oral hygiene behavior.

## Introduction

Indonesian Basic Health Research Data states that the prevalence of the Indonesian population experiencing dental and oral health problems has increased sharply from 25.9% in 2013 to 57.6% in 2018. 1 2 The data also identified one of the vulnerable groups for dental and oral problems which are adolescents aged between 10 and 14 where 55.6% suffer from dental and oral diseases. 3 4 Based on the 2018 Riskesdas data in West Java Province, the proportion of oral health problems in the age group of 10 to 14 years was found to be 8.28%, and the proportion of oral health for all age groups in Depok City reached 9.65%. The proportion of dental health problems in the age group of 10 to 14 years in West Java was 19.1%, and in the city of Depok, it reached 22.6%. 2

Entering early adolescence, a person experiences physical, mental, and psychological changes. Once the permanent teeth have completely erupted, and if the permanent teeth are damaged or lost, there is no replacement. Physical attractiveness in the adolescent phase is essential in socializing, which ultimately results in dissatisfaction if the appearance of his face, including parts of the mouth such as imperfect teeth. 5

Patterns of behavior during early adolescence play an essential role in maintaining oral hygiene because poor oral hygiene behavior will tend to continue into adulthood. 6 7 8 In adolescence, peer relationship is critical in everyday life, so they need to spend much time with friends. 9 The increased risk of oral health problems in adolescence can be caused by environmental influences such as peer association patterns in trying new things that can affect oral health, such as smoking and alcohol consumption. 10 Furthermore, the freedom to consume sugary foods and disregarding parental rules, may lead to neglecting oral hygiene habits like brushing teeth as a priority. 6 11 12 Thus, good behavior regarding oral hygiene should be formed as early as possible and starting in the family environment, especially among parents. 5 7 The role of parents, especially mothers, is vital for guiding, providing understanding, and providing facilities for maintaining children's oral health at home.

In parenting between parents and adolescents, the mother is a house member who has an excellent opportunity to create more intense care and closeness to adolescents in the family sphere. 13 Mothers are the primary caregivers of children from a young age, so knowledge, attitudes, and oral hygiene practices by mothers directly related to children's oral health become the first example for children in maintaining oral hygiene. This study aimed to determine the role of maternal parenting style in predicting oral hygiene behavior in adolescents aged between 12 and 14 years. It is hypothesized that a mother's parenting style affects adolescents' oral hygiene behavior. This study is essential, considering that adolescence is a period of changing behavior that can be carried over into adulthood. Hence, the results of this study are expected to provide supporting data in making health behavior programs for adolescents and their parents.

## Materials and Methods

Depok City, the location of this study, is in West Java Province, Indonesia. The city is divided into 11 districts with a total population of 180,941 early adolescents. The Pancoran Mas subdistrict was selected as the study location based on random selection. This subdistrict has 34 junior high schools, and two schools were chosen as study locations. This study has received approval from the Research Ethics Commission of Universitas Padjadjaran with document number 645/UN6.KEP/EC/2022. The type of research used is quantitative research with a cross-sectional approach. The study was conducted at two junior high schools in Depok (SMP PGRI Depok and SMP Negeri 11 Depok) in November 2022. The total population of the two schools is 250 students. Based on the correlative analytic formula, the sample size obtained a minimum sample size of 93 people. The sampling technique used total sampling on SMP PGRI students and multistage random sampling on students at SMP Negeri 11 Depok. Adolescents aged between 12 and 14 years whom their mothers had raised since childhood were included. Parents signed written consent prior to the study.

### Measurement of Mother's Parenting Style

Mother's parenting style is the behavior of mothers toward their children that is most prominent in caring for their children in everyday life. Parenting style was measured using a questionnaire adapted from parent as social context questionnaire, 14 which has been translated and adapted into Indonesian. The instrument consists of 24 items and is measured with a four-point Likert scale, 1 = very inappropriate to 4 = very suitable. This questionnaire has been tested for Indonesian respondence with a Cronbach alpha value of 0.70. 15 Scoring is done by combining scores for positive dimensions (warmth, structure, autonomy support) as supportive parenting and negative dimensions (rejection, chaos, coercion) as nonsupportive parenting.

### Measurement of Oral Hygiene Behavior

Oral hygiene behavior was measured using the oral hygiene behavior questionnaire based on the theory of planned behavior 16 and was adopted from Mahriani et al. 17 The measurement instrument encompasses attitudes towards behavior, subjective norms, behavioral control, and behavioral intentions. The questionnaire utilized two scales, the Likert scale and the semantic differential scale, to measure nineteen items. Oral hygiene behavior was assessed using ordinal scale data.

The attitudes dimension was evaluated using the semantic differential scale with four points ranging from 1=difficult to 4=easy, 1=unpleasant to 4=pleasant, and so on for pairs such as useless-useful, bad-good, not useful-useful, unhealthy-healthy, boring-not boring. The assessment of subjective norms, behavioral control, and intentions were measured using a four-point Likert scale with options ranging from 1=strongly disagree to 4=strongly agree.

All questionnaires were in the form of a written paper and distributed to each respondent to be filled out with an average filling time of 15 minutes. Previously, the researcher visited each class and gave directions to the respondents on how to fill out the questionnaire. Then, the researcher read and explained each question in the questionnaire so that each class could fill it out together.

### Data Analysis

The data were evaluated using the SPSS application, and data analysis in this study used a linear regression test to determine the role of maternal parenting style in predicting adolescent oral hygiene behavior. A frequency analysis was also carried out.

## Result

The total number of subjects who participated in this study was 230 adolescents. Table 1 shows that the respondents comprised 122 (53%) males and 108 (47%) females. Based on the age characteristics of the respondents, it was dominated by 14 years of age (39.6%) respondents. In addition, the respondent's level of education was dominated by grade 8, as many as 86 (37.4%) people.

**Table 1:** Participant characteristics

Characteristics	*n*	%
Gender		
Boys	122	53
Girls	108	47
Age (year)		
12	51	22.2
13	88	38.3
14	91	39.6
Grade		
7	80	34.8
8	86	37.4
9	64	27.8

Table 2 describes the various dimensions of mothers' supportive and nonsupportive parenting styles as reported by adolescents. In supportive parenting styles, adolescents dominantly agree that mothers have warmth (52.7%), regularity (54.1%), and autonomy support (56%). Meanwhile, nonsupportive parenting styles show that dominant adolescents disagree with their mothers, having characteristics originating from the rejection (47.6%) and chaos (43.4%) dimensions. Meanwhile, the coercion dimension's responses to agree and disagree were almost the same (38.8 vs. 35.2%).

**Table 2:** Mother's parenting style

**Supportive parenting**				
** Parenting style dimensions**	**Response,** ***n*** **(%)**			
	** Strongly disagree**	**Disagree**	**Agree**	**Strongly agree**
** 1. Warmth:** a. Mother showed her love for me b. mom enjoyed her time with me c. Mother always feels happy when she meets me d. Mom thinks I'm special	1 (0.4) 4 (1.7) 2 (0.9) 7 (3.0)	9 (3.9) 10 (4.3) 9 (3.9) 14 (6.1)	97 (42.2) 128 (55.7) 121 (52.6) 139 (60.4)	123 (53.5) 88 (3.3) 98 (42.6) 70 (30.4)
Subtotal	14 (1.5)	42 (4.6)	485 (52.7)	379 (41.2)
** 2. Structure:** a. Mother shows the way and gives guidance b. Mother gives an explanation about something c. My mother accompanies me to find solutions to the problems faced d. My mother explained the reasons for the rules that apply in the family	3 (1.3) 1 (0.4) 4 (1.7) 6 (6.5)	15 (6.5) 22 (9.6) 22 (9.6) 9 (9.7)	109 (47.4) 121 (52.6) 122 (53.0) 59 (63.4)	103 (44.8) 86 (37.4) 82 (35.7) 19 (20.4)
Subtotal	14 (2.5)	68 (8.9)	411 (54.1)	290 (34.6)
** 3. Autonomy support:** a. Mother trusts me b. Mother accepts me as I am c. Mother allowed me to do something important d. Mom tries to understand my point of view	- - 4 (1.7) 7 (3.0)	5 (2.2) 6 (2.6) 11 (4.8) 27 (11.7)	131 (57.0) 99 (43.0) 124 (53.9) 161 (70.0)	94 (40.9) 125 (54.3) 91 (39.6) 35 (15.2)
Subtotal	11 (1.2)	49 (5.3)	515 (56.0)	345 (37.5)
**Nonsupportive parenting**				
** Parenting style dimensions**	**Response,** ***n*** **(%)**			
	**Strongly disagree**	**Disagree**	**Agree**	**Strongly agree**
** 1. Rejection:** a. Sometimes I wonder if my mother likes me b. My mother always thought that I was getting in her way c. My mother made me feel unwanted d. Mother is always dissatisfied at what I do	15 (6.5) 64 (27.8) 84 (36.5) 45 (19.6)	59 (25.7) 131 (57.0) 119 (51.7) 129 (56.1)	120 (52.2) 29 (12.6) 22 (9.6) 46 (20.0)	36 (15.7) 6 (2.6) 5 (2.2) 10 (4.3)
Subtotal	208 (22.6)	438 (47.6)	217 (23.6)	57 (6.2)
** 2. Chaos:** a. When my mother made an appointment, I didn't know if she would keep her promise b. when my mother said she would do something, sometimes she didn't c. mom I fiddled with the rules on me d. My mother suddenly got mad at me for no reason	8 (3.5) 16 (7.0) 27 (11.7) 65 (28.3)	73 (31.7) 102 (44.3) 118 (51.3) 106 (46.1)	125 (54.3) 97 (42.2) 68 (29.6) 45 (19.6)	24 (10.4) 15 (6.5) 17 (7.4) 14 (6.1)
Subtotal	116 (12.6)	399 (43.4)	335 (36.4)	70 (7.6)
** 3. Coercion:** a. My mother dictated what to do b. My mother is my boss c. My mother thinks there is only one right way – her way d. My mother says “no” to everything	14 (6.1) 22 (9.6) 13 (5.7) 79 (34.3)	49 (21.3) 69 (30.0) 83 (36.1) 123 (53.5)	133 (57.8) 98 (42.6) 105 (45.7) 21 (9.1)	34 (14.8) 41 (17.8) 29 (12.6) 7 (30)
Subtotal	128 (13.9)	324 (35.2)	357 (38.8)	111 (2.1)

The oral hygiene behavior of adolescents can be seen in Table 3 . In the attitude dimension, the dominant adolescents gave a positive response (53.4%) in which 49.6% adolescents said that maintaining oral hygiene by brushing their teeth twice a day is easy, 71.7% said fun, 62.6% said not useless, 49.9% said good, 57.8% said useful, 51.7% said healthy, and 67.4% said not dull. For the subjective norm dimension, the presence of mothers (53.5%), teachers (60.9%), and other relatives (50%) who order them to brush their teeth twice a day is the norm that influences their behavior. Finally, in the behavioral control dimension, the dominant adolescents gave a response that agreed that they could control their brushing behavior (44.8%), thus leading to their intention to behave that way (57.5%). The total score of adolescent oral hygiene behavior is 60.37 ( Table 4 ).

**Table 3:** Adolescent oral hygiene behavior

**Score,** ***n*** **(%)**				
	**1**	**2**	**3**	**4**
** 1. Attitude “maintain oral hygiene by brushing teeth 2x a day”** a. Difficult—easy b. Not fun—fun c. Useless—not useless d. Bad—good e. Not useful—useful f. Unhealthy—healthy g. Boring—not boring	- 2 (0.9) 1 (0.4) 2 (0.9) 1 (0.4) - 2 (0.9)	7 (3.0) 17 (7.4) 4 (1.7) 11 (4.8) 7 (3.0) 2 (0.9) 27 (11.7)	114 (49.6) 165 (71.7) 81 (35.2) 103 (44.8) 133 (57.8) 109 (47.4) 155 (67.4)	109 (47.4) 46 (20.0) 144 (62.6) 114 (49.6) 89 (38.7) 119 (51.7) 46 (20.0)
**Subtotal**	8 (0.5)	75 (4.6)	860 (53.4)	667 (41.5)
**Score,** ***n*** **(%)**				
	**Strongly disagree**	**Disagree**	**Agree**	**Strongly agree**
2. Subjective norms a. Mother told me to brush my teeth 2x a day b. The teacher told me to brush my teeth 2x a day c. Friends told me to brush my teeth twice a day d. Someone else (uncle/aunt/sister or other) told me brush my teeth 2x a day	– 6 (2.6) 44 (19.1) 8 (3.5)	13 (5.7) 57 (24.8) 148 (64.3) 100 (43.5)	123 (53.5) 140 (60.9) 34 (14.8) 115 (50.0)	94 (40.9) 27 (11.7) 4 (1.7) 7 (3.0)
**Subtotal**	58 (6.3)	318 (34.6)	412 (44.8)	132 (14.3)
3. Behavior control: a. If I want, I can brush my teeth 2x a day b. For me, brushing my teeth 2x a day is easy c. I can brush my teeth 2x a day d. Brushing my teeth 2x a day is easy for me	6 (2.6) – 2 (0.9) 5 (2.2)	23 (10.0) 9 (3.9) 12 (5.2) 21 (9.1)	133 (57.8) 127 (55.2) 146 (63.5) 116 (50.4)	68 (29.6) 94 (40.9) 70 (30.4) 88 (38.3)
**Subtotal**	13 (1.4)	65 (7.1)	522 (56.7)	320 (34.8)
4. Behavior intension: a. I will routinely brush my teeth twice a day every day b. I am willing to brush my teeth twice a day every day c. I intend to brush my teeth twice a day every day d. I want to brush my teeth twice a day every day	1 (0.4) – – 2 (0.9)	12 (5.2) 20 (8.7) 13 (5.7) 12 (5.2)	136 (59.1) 128 (55.7) 136 (59.1) 129 (56.1)	81 (35.2) 82 (35.7) 81 (35.2) 87 (37.8)
**Subtotal**	3 (0.3)	57 (6.2)	529 (57,5)	331 (36.0)

**Table 4:** A total score of adolescent oral hygiene behavior

		**Statistical measure**		
**Subvariable**	**Mean**	**Standard deviation**	**Max**	**Min**
• Intension • Attitude toward behavior • Subjective norms • Behavior control	13,17 23,51 10,69 13,00	1,887 2,579 1,574 1,709	16 28 15 16	8 15 6 8
Total score of adolescent oral hygiene behavior	60,37	5,730	73	44

The results of the correlation test in Table 5 regarding the correlation of the role of a mother's parenting style with attitudes, subjective norms, behavioral control, intention, and total adolescent oral hygiene behavior using the Spearman rank test. The correlation coefficient ( *r* ) was obtained for the total nonsupportive parenting style of −0.063 ( *p* = 0.338) and the supportive parenting style of 0.558 ( *p* = 0.000).

**Table 5:** Correlation of mother's parenting style with oral hygiene behavior subvariables

**Attitude toward behavior**	***r***	***p*** **-Value**	**Result**
**Supportive** Warmth Structure Autonomy support **Nonsupportive** Rejection Chaos Coercion	0.502 0.443 0.375 0.364 − 0.022 − 0.080 − 0.023 0.046	0.000 0.000 0.000 0,000 0,742 0.225 0.731 0.491	Significant Significant Significant Significant Not significant Not significant Not significant Not significant
**Subjective norms**			
**Supportive** Warmth Structure Autonomy support **Nonsuportive** Rejection Chaos Coercion	0.282 0.218 0.234 0.213 − 0.024 − 0.036 − 0.069 0.142	0.000 0.001 0.000 0.001 0.719 0.586 0.299 0.031	Significant Significant Significant Significant Not significant Not significant Not significant Significant
**Behavior control**			
**Supportive** Warmth Structure Autonomy support **Nonsuportive** Rejection Chaos Coercion	0.487 0.392 0.369 0.394 − 0.118 − 0.159 − 0.041 0.071	0.000 0.001 0.000 0.001 0.073 0.016 0.538 0.285	Significant Significant Significant Significant Not significant Significant Not significant Not significant
**Intention**			
**Supportive** Warmth Structure Autonomy support **Nonsuportive** Rejection Chaos Coercion	0.333 0.263 0.222 0.316 − 0.75 − 0.119 − 0.035 0.021	0.000 0.000 0.001 0.000 0.254 0.071 0.593 0.754	Significant Significant Significant Significant Not significant Not significant Not significant Not significant
**Total oral hygiene behavior**			
**Supportive** Warmth Structure Autonomy support **Nonsupportive** Rejection Chaos Coercion	0.558 0.463 0.416 0.444 − 0.063 − 0.133 − 0.053 0.032	0.000 0.000 0.000 0.000 0.338 0.044 0.424 0.632	Significant Significant Significant Significant Not significant Significant Not significant Not significant

*r* = Spearman rank correlation coefficient.

The regression analysis conducted in Tables 6 and 7 examined the impact of maternal parenting style on adolescent oral hygiene behavior using multiple regression tests. The regression coefficient of nonsupportive parenting style was 0.044 (sig=0.567), while the coefficient of supportive parenting style was 0.Q9 Q9785 (sig=0.000). The level of contribution made by supportive parenting style to adolescent oral hygiene behavior was revealed by the calculated determinants, which resulted in an R2 value of 31.2%.

**Table 6:** Effect of nonsupportive and supportive parenting styles on oral hygiene behavior

**Variable**	**Coefficient regression**	**Error standard**	***t***	**Sig.**
**Konstanta** Nonsupportive Supportive Warmth Structure Autonomy support	30,726 − 0.044 0.785 0.882 0.569 0.975	3 **,** 863 0.077 0.078 0.197 0.195 0.259	7,953 -0.573 10.096 4,483 2,912 3,763	0.000 0.567 0.000 0.000 0.004 0.000

**Table 7:** Coefficient of determination of supportive parenting style on adolescent oral hygiene behavior

**Variable**	***r***	***r*** **2**	**Adjusted** ***r*** **Square**	**Standard error**
Supportive parenting	0.558	0.312	0.309	4,764

From the calculation of Spearman rank correlation, it is known that parenting style, which is significantly related to the formation of adolescent oral hygiene behavior, is all dimensions of supportive parenting style consisting of warmth, structure, and autonomy support as well as rejection dimensions that are included in the dimension of nonsupportive parenting style. In the influence test with regression, only the dimensions of supportive parenting consisting of warmth, structure, and autonomy support significantly affected adolescent oral hygiene behavior by 31.2%. This shows that the supportive parenting style variable contributes to adolescent oral hygiene behavior by 31.2%, while other variables outside this study influence the remaining 68.8%.

## Discussion

Parenting that was applied by mothers to adolescents, as reported by students at SMP PGRI and SMP Negeri 11 Depok, showed that the supportive parenting style held a more significant percentage than the nonsupportive parenting style. Based on the study's results, the highest percentage of parenting style obtained was the warmth dimension, with an average total percentage of adolescents agreeing and strongly agreeing about the statement from the warm parenting style dimension reaching 46.95%. The lowest percentage of parenting style obtained was the rejection dimension, with an average total percentage of adolescents agreeing and strongly agreeing with statements from the dimension of rejection is 14.9%. In Abidin et al.'s study, it was also stated that the warmth dimension had the highest average score of other supportive parenting style dimensions in Indonesia. 15

Maternal parenting is the most dominant parenting style that mothers apply to children from a young age in supporting children's physical, social, emotional, intellectual and spiritual development. Every parent has different parenting styles for educating children. Various parenting styles are influenced by education, ethnicity and culture, socioeconomic families, and the history of parenting styles experienced by these parents. 18

The most frequently chosen statement by respondents on the warm dimension of supportive parenting style is “my mother shows her love for me,” with a total percentage of agree-strongly agree reaching 95.7%. Children with the warm dimension parenting style will feel more accepted, valued and cared for by the mother. 14 The application of the warmth dimension allows children to be close and open to their mothers. 19 This allows children to adapt oral hygiene maintenance behaviors taught by mothers from childhood and can be followed routinely without any coercion felt by the child. 5

Based on Tables 5 to 7 , it is known that there is a significant relationship and influence between the mother's supportive parenting style and the total oral hygiene behavior of adolescents, consisting of intentions, attitudes, behavioral controls, and subjective norms. Based on the classification of the closeness of the relationship between variables using Guilford's Criteria (1956), 20 supportive parenting style has a reasonably close relationship with adolescent oral hygiene behavior variables. From the linear regression results, each parenting dimension has a significant effect with a positive direction regression coefficient on oral hygiene behavior. This data shows that higher the application of the dimensions of supportive parenting style, such as warmth, structure, and autonomy support by the mother, the better the formation of adolescent oral hygiene behavior.

Mothers with warm, regular, and autonomous support will create an environment that provides affection, enforces discipline, and clear rules and provides good facilities so that good oral hygiene behavior is formed because children feel confident and capable and find it easy to perform oral hygiene—supported by their environment. This statement is in line with Fitri's study, which states that health behavior is determined by whether or not the provision of facilities and infrastructure is a supporting factor. 21 Zakiudin's research states that there is a significant relationship between the existence of clear regulations regarding personal hygiene and the personal hygiene behavior of students at the Brebes Islamic Boarding School. 22

The results of this study were also supported by the study of Wanti et al, which stated that there was an influence of extrinsic motivation in the form of the role of parents in maintaining children's dental health. 23 Assistance provided by mothers can change children's behavior and have a higher level of motivation in behavior, including oral hygiene behavior. Hamida's research shows that the better the parenting style applied to children, the better the child's level of independence. 24

In nonsupportive parenting, the coercion dimension has the highest total average percentage in the agree-strongly agree column. This finding states that coercion is the negative way mothers dominate raising children. Respondents' most frequently chosen statement in the coercion subdimension was, “my mother dictated what I should do”. In the correlation test, the dimension of coercion had a weak positive significant relationship with subjective norms and had no relationship with overall oral hygiene behavior. Subjective norms refer to individual subjective judgments about how others view this behavior. 7 Mothers who tend to apply coercive parenting to their children will control them excessively, limiting children and demanding that children obey the rules made by parents. 14 Children will view oral hygiene behavior as necessary thing that must be carried out, and the child must implement this behavior by the mother. This study's application of subjective norm factors in forming oral hygiene behavior has a minor average of the other factors. Bramantoro et al state that subjective norms are one of the adolescents' weakest predictors of tooth brushing behavior. 25

In the rejection dimension, there is a significant negative relationship between behavioral control factors and total adolescent oral hygiene behavior. The higher the application of refusal parenting by the mother, the lower the control of the child's oral hygiene behavior and behavior. Behavioral control refers to a person's judgment about how likely it is to carry out a behavior based on the results of an evaluation of that behavior. If a person believes and can clean his mouth by brushing his teeth regularly, he will tend to have the intention to clean his mouth by brushing his teeth twice a day regularly. In the rejection dimension, mothers tend to show reluctance and refuse when children ask for help which causes children to find it difficult to practice good oral hygiene behavior. 14 15

The results of this study are in contrast to the research of de Jong et al, which stated that ineffective parenting characterized by disciplinary inconsistencies and excessive demands could lead to low levels of child compliance and harm children's adherence to brushing their teeth twice a day. 26 The influence of differences in the target sample of the research was in filling out the questionnaire because, in that study, the sample was parents with school-age children, while in this study, the sample was teenagers who had experienced the development of their intellectual abilities and independence. Adolescents generally have high levels of delinquency and problematic behavior and will not obey parents who care for them with a negative parenting style. 27 28

According to the theory of planned behavior, the formation of behavior is determined by one's intention, which is a combination of attitudes, subjective norms, and perceived behavioral control. Each individual has various variables to encourage them to do some behavior. Different experiences and levels of knowledge can cause this difference. 26 When an individual has a positive attitude towards a behavior, perceiving it as having a positive impact and being easy/fun to do, and also feels pressure/encouragement from others to perform the behavior, along with believing they have the ability and resources to support the behavior, their intention to perform the behavior (such as oral hygiene behavior) increases. This, in turn, results in the individual carrying out the oral hygiene behavior. 29

Mothers have a central role in maintaining their children's health and are an essential social model in forming dental and oral hygiene behavior in adolescence because this behavior will be carried over into adulthood. Other studies have also stated that mothers' knowledge about oral health significantly affects the oral health behavior of their adolescent children. 30 From these various studies, it is proven that mothers have an essential role in promoting positive attitudes towards oral hygiene behavior in their children from childhood to adolescence, so parenting is needed. Maternal support such as warmth, regularity, and autonomy support in creating good adolescent oral hygiene behavior.

There are limitations to this study that are as follows: the respondent variant is only in one domicile, there is a risk of biased answers because adolescents fill out the questionnaire using self-report, and the filling process is carried out together in class where there is a possibility of cheating even though instructions have been given and supervised by adults from the start. Future studies are expected to take more varied subjects with different characteristics and cultures from several regions. Consider distributing questionnaires to mothers in assessing mother-child care related to parenting carried out in the family and demographic data such as age, education, and mother's economy to see factors that influence parenting style.

## Conclusion

Based on the study's results, the mother's supportive parenting style predicts adolescent oral hygiene behavior at SMP PGRI Depok and SMP Negeri 11 Depok. The better the application of style supportive care that mothers do, the better the oral hygiene behavior of adolescents. However, there was no significant effect of the mother's nonsupportive parenting style on the oral hygiene behavior of adolescents at SMP PGRI Depok and SMP Negeri 11 Depok. Mothers with nonsupportive parenting styles do not help improve adolescent oral hygiene behavior. Therefore, mothers must apply a supportive parenting style to support the formation of good oral hygiene behavior in adolescents.
